# Anionic Species Regulate Chemical Storage in Nanometer Vesicles and Amperometrically Detected Exocytotic Dynamics

**DOI:** 10.1021/jacs.2c00581

**Published:** 2022-03-07

**Authors:** Xiulan He, Andrew G. Ewing

**Affiliations:** Department of Chemistry and Molecular Biology, University of Gothenburg, 412 96 Gothenburg, Sweden

## Abstract

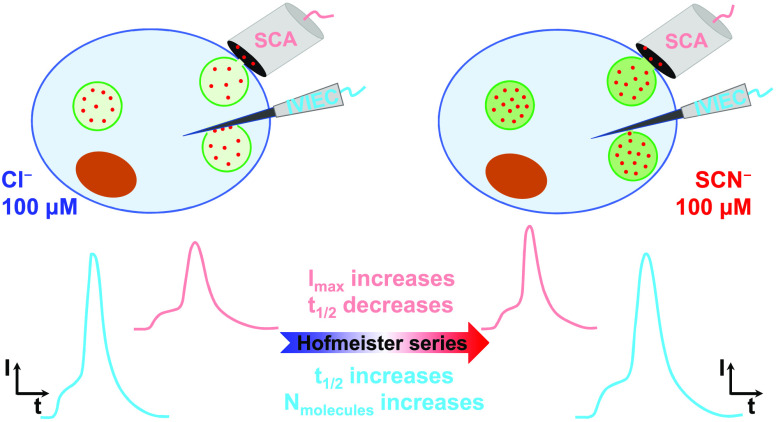

Hofmeister effects have often been ignored in living organisms, although they affect the activity and functions of biological molecules. Herein, amperometry has been applied to show that the vesicular content, dynamics of exocytosis and vesicles opening, depend on the anionic species treatment. Compared to 100 μM Cl^–^ treated chromaffin cells, a similar number of catecholamine molecules is released after chaotropic anions (ClO_4_^–^ and SCN^–^) treatment, even though the vesicular catecholamine content significantly increases, suggesting a lower release fraction. In addition, there are opposite effects on the dynamics of vesicles release (shorter duration) and vesicle opening (longer duration) for chaotropic anions treated cells. Our results show anion-dependent vesicle release, vesicle opening, and vesicular content, providing understanding of the pharmacological and pathological processes induced by inorganic ions.

The Hofmeister series is the earliest specific ion effect, known for more than a century.^1^ It can be divided into anionic and cationic series and ordered by their ability to precipitate proteins from solution: CO_3_^2–^ > SO_4_^2–^ > H_2_PO_4_^–^ > F^–^ > Cl^–^ > Br^–^ > NO_3_^–^ > ClO_4_^–^ > SCN^–^, and NH_4_^+^ > Cs^+^ > K^+^ > Na^+^ > Li^+^ > Ca^2+^ > Mg^2+^ > Zn^2+^. It has been studied in biological systems (e.g., proteins,^2,3^ enzymes,^4,5^ biochannels,^6^ and lipids^7,8^), but Hofmeister effects have often been ignored in living organisms, although they are generally based on various aqueous salts including numerous anions and cations, which are listed in the Hofmeister series. For anions, ClO_4_^–^, SCN^–^, and NO_3_^–^ can act as inhibitors of iodide transport which might affect the function of the thyroid,^9,10^ ClO_4_^–^ also can affect the release of insulin,^11^ and Br^–^ has been applied as a drug to treat epilepsy.^12,13^ Hofmeister series anions have been reported to affect in vitro aggregation of several amyloidogenic proteins, which are involved in the onset of neurodegenerative disease.^14−18^ Recently, we observed that counteranions in the stimulation solution alter the dynamics of exocytosis that can be explained by immediate Hofmeister effects on lipid bilayers of the cell membrane.^19^ Of particular interest, however, is the investigation of the Hofmeister effect in living biological models (e.g., cell, tissue, and animal).

In this paper, chromaffin cells were treated with 100 μM KX (X^–^: Cl^–^, Br^–^, NO_3_^–^, ClO_4_^–^, SCN^–^) for 3 h before amperometric methods. We used intracellular vesicle impact electrochemical cytometry (IVIEC) to detect the opening of vesicles on the electrode in the intracellular environment, and simultaneously applied single cell amperometry (SCA) to monitor the exocytotic process at cells triggered with 30-s 30 mM KCl stimulation solution (see Supporting Information S1). Combined with these two methods, we quantified the catecholamines stored in vesicles and compared them to exocytotic release, calculating the fraction of catecholamine released in each event. In addition, the high temporal resolution of amperometry allows the dynamics of vesicle release and vesicle opening to be measured.

Typical IVIEC amperometric traces for vesicle opening are shown in Figure 1A–B for Cl^–^ and SCN^–^ treated, and in Figure S1 for Br^–^, NO_3_^–^, and ClO_4_^–^ treated, chromaffin cells. The number of molecules (*N*_molecules_) in each vesicle can be quantified from each current transient by Faraday’s law. A log-normalized frequency histogram of the molecular count in each vesicle is shown in Figure 1C. A near-Gaussian distribution with similar standard deviation but different mean values is observed for each distribution. We also compared the medians of *N*_molecules_ per vesicle obtained across the Hofmeister series (Figure 1D), where the vesicular catecholamine content is observed to increase significantly after the exposure of the chaotropic anions, ClO_4_^–^ and SCN^–^ (*p* values are listed in Table S1). Vesicular catecholamine content is dominated by two competing pathways: the catecholamine transport into the vesicles by the vesicular monoamine transporter and extracellular inactivation by monoamine oxidases and catechol-*O*-methyltransferase.^20,21^ We assume that the increased vesicular content might be induced by the inhibition of enzymes via the chaotropic effect. Chaotropic anions have been shown to affect catecholamine transport across the vesicular membrane, membrane potential, and intravesicular pH,^22−24^ which will affect the vesicular amine concentration.

**Figure 1:**
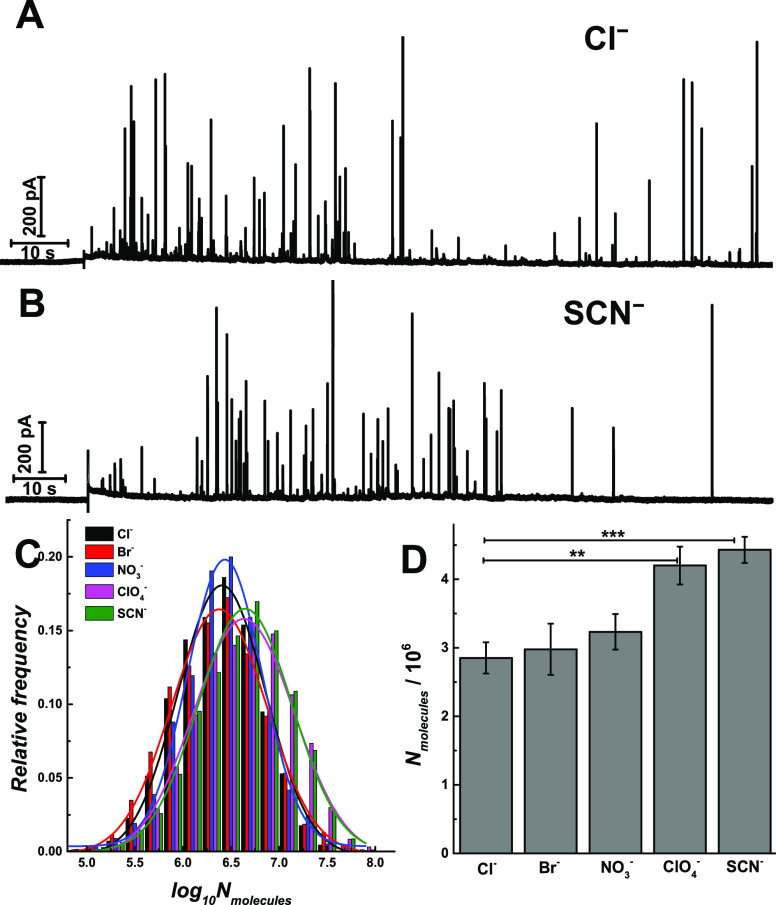
IVIEC amperometric traces obtained from chromaffin cells treated with 100 μM KCl (A) and KSCN (B) for 3 h, respectively. (C) Normalized frequency histograms describing the distributions of the log_10_*N*_molecules_ obtained from IVIEC of chromaffin cells which were treated with different anions. (D) Comparisons of *N*_molecules_. Pairs of data sets were compared with *t* test; ***, *p* < 0.001; **, *p* < 0.01, *n* = 12.

Typical SCA amperometric traces of exocytosis from chromaffin cells are shown in Figure 2A–B after exposure of the cells to Cl^–^ or SCN^–^ and in Figure S2 for Br^–^, NO_3_^–^, or ClO_4_^–^. Upon stimulation, the vesicle membrane fuses with the cell membrane and releases part of the vesicle content, which is recorded as a trace of current transients representing exocytotic release events. The number of release events (*N*_events_) was calculated for each condition (Figure S3A). Compared to Cl^–^, the *N*_events_ decreases significantly when the cells were treated with SCN^–^ (*p* values are listed in Table S2). Similarly, a log-normalized frequency histogram of *N*_molecules_ after the exposure of each anion is shown in Figure 2C, for which a near-Gaussian distribution and similar standard deviation are again observed but varied mean values are observed for the different anions treated chromaffin cells. Furthermore, the mean values of the average *N*_molecules_ released from chromaffin cells after the exposure to the different anions is also compared and shown in Figure 2D (*p* values in Table S3). There is no significant difference between the release molecule count after the exposure of kosmotropic anions (Cl^–^) and chaotropic anions (ClO_4_^–^, SCN^–^). Interestingly, a decrease in the number of molecules released is observed after the exposure of Br^–^, whereas an increase in molecules released is observed after exposure to NO_3_^–^. As the molecular count for catecholamine release and the vesicle content are both obtained here, the release fraction can be calculated (Figure S3B). Comparing the fraction released after the exposure of kosmotropic anions (Cl^–^, 42%), we found a smaller release fraction for chaotropic anions (ClO_4_^–^, 29%; SCN^–^, 27%). Interestingly, Br^–^ decreases the release and release fraction (36%), which might reverse the seizure activity and help to understand the mechanism of epilepsy treatment.^25^

**Figure 2:**
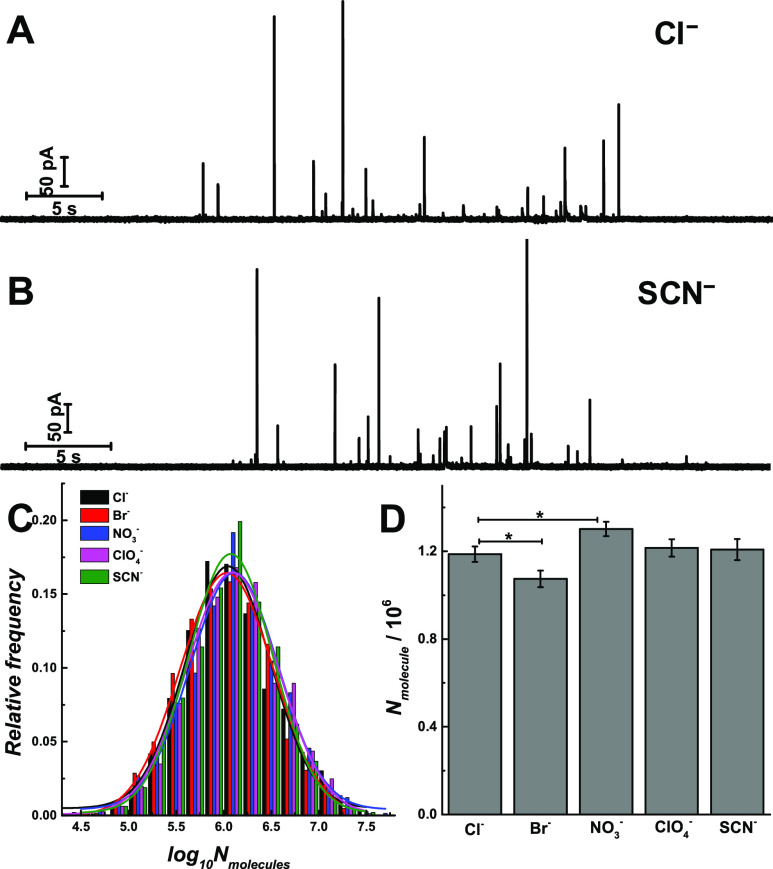
SCA amperometric traces obtained from chromaffin cells which were stimulated by 30-s 30 mM KCl solution after treatment of 100 μM KCl (A) and KSCN (B) for 3 h, respectively. (C) Normalized frequency histograms describing the distributions of the log_10_*N*_molecules_ obtained from SCA of chromaffin cells which different anions. (D) Comparisons of *N*_molecules_. Pairs of data sets were compared with *t* test; *, *p* < 0.05, *n* = 30.

The release dynamics are clearly important in controlling the release fraction for partial release.^26,27^ We analyzed the dynamic parameters (Figure S4) of current spikes in SCA amperometric traces, including *I*_max_, the peak amplitude, *t*_1/2_, the half peak width, *t*_rise_, the rise time, and *t*_fall_, the fall time, and compared them in Figure 3 (*p* values are listed in Tables S4–S7). Exocytosis at treated chromaffin cells with different anions displays a larger *I*_max_ as the anion is changed from kosmotropic to chaotropic anions (Figure 3A), which agrees with the enrichment of vesicle content caused by chaotropic anions. However, a smaller value of *t*_rise_, *t*_1/2_, and *t*_fall_ was observed (Figure 3B–D) for exocytosis after exposure of chaotropic anions, suggesting a faster opening, shorter duration, and faster closing of fusion pore, showing lower durability of the fusion pore than after exposure of kosmotropic anions.

**Figure 3:**
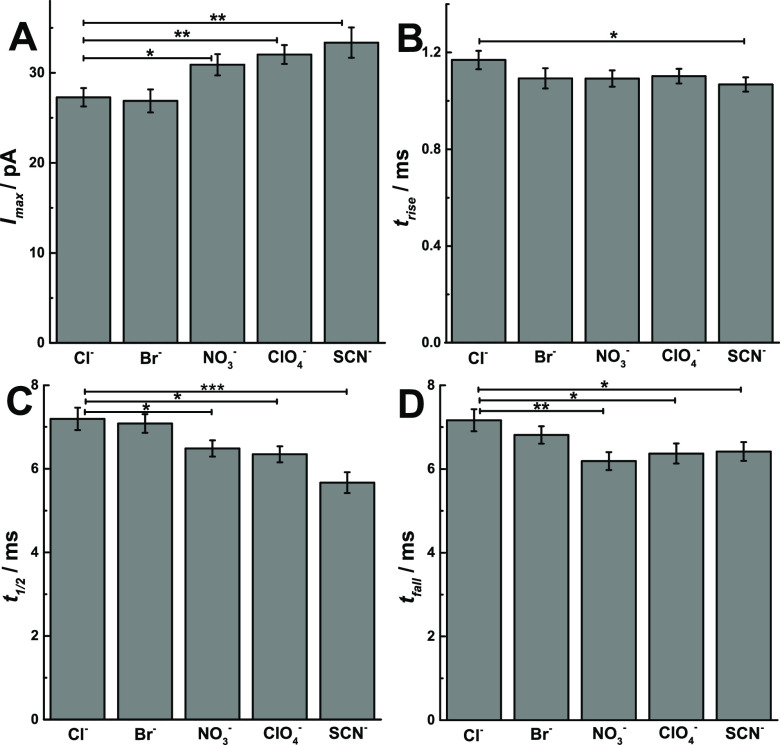
Scheme showing the peak analysis, comparisons of (A) *I*_max_, (B) t_rise_, (C) *t*_1/2_, and (D) *t*_fall_, obtained from SCA of chromaffin cells which were stimulated by 30-s 30 mM KCl solution after treatment of different anions. Pairs of data sets were compared with *t* test; *** *p* < 0.001; ** *p* < 0.01; * *p* < 0.05, *n* = 30. Events per cell/treatment are listed in Figure S3.

An opposite effect in exocytotic dynamics has been observed in chromaffin cells, which is induced by the chaotropic effects on the lipids bilayer.^19^ The different Hofmeister effects might be induced by chaotropic interactions with different biological molecules that are related to exocytosis, helping to further understand the Hofmeister series in cell biology. In addition to phospholipids,^28,29^ proteins (soluble *N*-ethylmaleimide sensitive factor attachment protein receptors complex proteins (SNAREs),^30^ actin,^31,32^ and dynamin^32,33^) and enzymes (e.g., protein kinase C (PKC))^34^ regulate exocytosis at the cell membrane. Anions can interact with proteins via ion-pairing, hydrogen bonding, π-anion, and chaotropic effects.^3,35−37^ The chaotropic anions prefer to interact with the hydrophobic regions of proteins directly modifying the activity and increasing solubility of proteins. However, kosmotropic anions are thought to interact with proteins via mediating water molecules. In this case, chaotropes (SCN^–^) help to unfold proteins and induce a salting-in effect. By comparison, kosmotropes (Cl^–^) lead to the stabilization of the folded state and cause a salting-out effect. Therefore, it seems plausible that chaotropic anions can increase the formation of the SNARE complex in the cell membrane by decreasing the protein–protein interaction between SNAREs and other noncognate SNAREs,^38,39^ resulting in a shorter duration. Moreover, the activity of PKC can be inhibited by chaotropic anions via increasing its solubility because it is water-soluble in the inactive state.^40,41^ As a result, the activation of actin fragmentation causes a tighter actin network which results in a less stable fusion and shorter exocytotic events before the pore closes again.^42,43^ The loosening assembly around microtubules of dynamin induced by adsorption of chaotropic anions might lead to a shorter duration of exocytosis events.^44^ Interestingly, the effect of chaotropic anions contrasts the effects of Zn^2+^ (kosmotropes).^45^ This is, again, consistent with the Hofmeister effect.

To support the proposed mechanism, we analyzed the parameters of prespike feet from SCA experiments to provide insights into the initial fusion pore after treatment with different anions, including *I*_foot_ and *t*_foot_ (Figure S4), representing the current amplitude and lifetime, respectively. The released molecules during the prespike foot can be calculated via the area of the foot part. Values for *I*_foot_, *t*_foot_, *N*_molecules_(foot), and *N*_foot_/*N*_events_ decrease significantly after the exposure of chaotropic anions (e.g., ClO_4_^–^) as shown in Figure S5 (*p* values are listed in Tables S8–S11). The value for *I*_foot_ decreases (ClO_4_^–^) or remains constant (SCN^–^) after exposure of chaotropic anions, even though the vesicle content increases simultaneously, suggesting a smaller initial pore,^46^ which could be induced by a tighter actin network than after exposure to Cl^–^. Moreover, the decreased *t*_foot_, *N*_foot_/*N*_events_, and *N*_molecules_ in the foot indicate that the chaotropic anions induce a less stable fusion pore, which is consistent with the results from the main peak. It could be induced by a tighter actin network and looser dynamin after interacting with chaotropic anions as mentioned in the proposed mechanism.

Peak parameters for vesicle opening obtained by IVIEC are summarized and analyzed in Figure 4 (*p* values are listed in Tables S12–S15). There is no significant difference observed between the values of *I*_max_ obtained for different anions treated cells (Figure 4A). However, slower opening and longer duration responses (e.g., larger *t*_rise_, *t*_1/2_, and *t*_fall_, shown in Figure 4B–D) were observed when the cells were treated with chaotropic anions (e.g., SCN^–^). This is opposite to the effect of chaotropic anions on the dynamics of exocytosis, but is consistent with the previous observation.^19^ We assume that this is due to the adsorption of chaotropic anions on the dense-core and vesicular lipid bilayer, and chaotropic anions affect the association of adrenaline with intravesicular matrix species (chromogranins, ATP, calcium) as their colligative properties are strongly dependent on pH,^47^ which leads to a slower vesicle opening event on the electrode. These results suggest the chaotropic anions affect different targets by varying their treatment mode, concentration, and time, which could be helpful to understand the chemical interactions and use to regulate release fraction.

**Figure 4:**
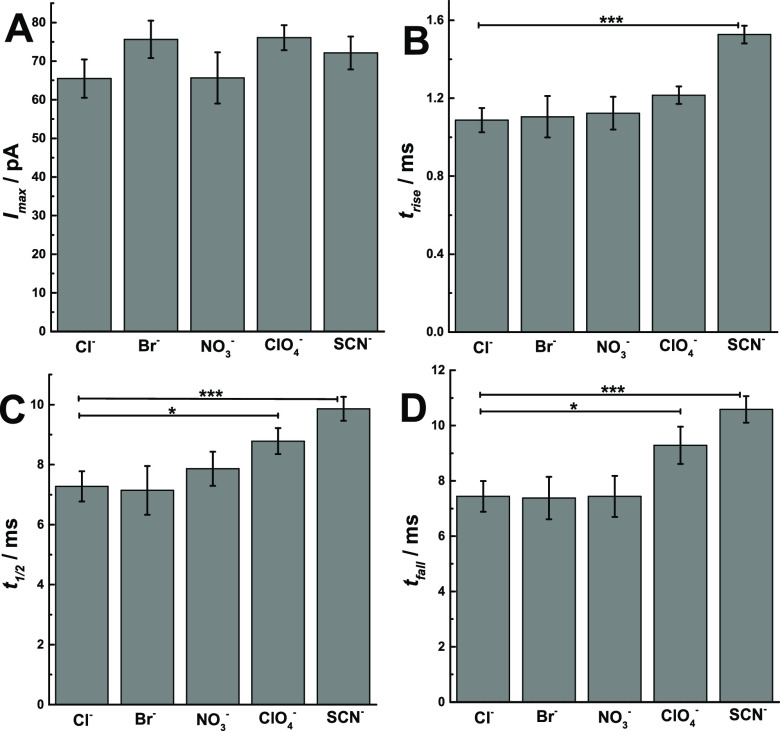
Scheme showing the peak analysis, comparisons of (A) *I*_max_, (B) t_rise_, (C) *t*_1/2_, and (D) *t*_fall_, obtained from IVIEC of chromaffin cells which were treated with 100 μM KX (X^–^: Cl^–^, Br^–^, NO_3_^–^, ClO_4_^–^, SCN^–^) for 3 h. Pairs of data sets were compared with *t* test; ***, *p* < 0.001; **, *p* < 0.01; *, *p* < 0.05, *n* = 12.

In conclusion, amperometric measurements show that micromolar concentrations of chaotropic anions not only trigger changes in the dynamics of exocytosis but also, importantly, vesicle content in secreting cells, a novel discovery. The vesicle catecholamine content in chromaffin cells is significantly increased after 3-h exposure to 100 μM chaotropic anions (e.g., ClO_4_^–^ and SCN^–^). However, catecholamine release during exocytosis remains nearly the same, resulting in a smaller release fraction which has been implicated as an important factor as part of regulated exocytosis in plasticity and cognition.^48−51^ Further, chaotropic anions shorten the duration time of the membrane fusion pore during exocytosis. We assume the chaotropic anions can affect the physicochemical properties of proteins via chaotropic effects, including an increase in the formation of SNAREs, decrease in the activity of PKC, tightening of the structure of the actin network, and loosening of the structure of dynamin, all resulting in a shorter exocytotic process. Our results suggest the process of vesicle release, and the amount vesicular content in living cells can be regulated by Hofmeister effects, which might be helpful to understand the many pharmacological and pathological processes induced by ions.

## Supporting Information Available

Chemical reagents, electrochemical measurements, data acquisition and analysis, isolation of adrenal chromaffin cells, fabrication of disk carbon fiber microelectrodes, fabrication of nanotip conical carbon fiber microelectrodes, Figures S1–S5, and Tables S1–S15. (PDF)
