# Supplementary material for: Anionic Species Regulate Chemical Storage in Nanometer Vesicles and Amperometrically Detected Exocytotic Dynamics
Source: J Am Chem Soc. 2022 Mar 7;144(10):4310–4. doi: 10.1021/jacs.2c00581 (PMC8931764; doi:10.1021/jacs.2c00581)
Supplement: Supplementary file 1 — ja2c00581_si_001.pdf [file ja2c00581_si_001.pdf]

Electronic Supplementary Information

Anionic Species Regulate Chemical Storage in Nanometer Vesicles and Amperometrically Detected Exocytotic Dynamics

Xiulan He, Andrew G. Ewing*

Department of Chemistry and Molecular Biology, University of Gothenburg, 41296 Gothenburg, Sweden.

Table of contents

Experimental Section……………………………………………..……..……..………S1
Figure S1…………………………..……..……………..……..………..…...….S5
Figure S2……………………………………………………………………..….S6
Figure S3 and Figure S4……………………………………………………………S7
Figure S5……………………………………………………………………………S8
Table S1 to Table S15……..……………………………………………...……….S9

S1. Experimental Section

1. Chemical Reagents: Chemicals and Solutions. Unless otherwise specified, all reagents were purchased from Sigma-Aldrich. All chemicals, of analytical grade, were obtained from Sigma-Aldrich and used as received. All solutions were prepared using 18 MΩ·cm water from Purelab Classic purification (ELGA, Sweden), adjusted to pH 7.4 and filtered prior for use. The isotonic saline solution included 150 mM NaCl, 5 mM KCl, 1.2 mM $MgCl_2$, 2 mM $CaCl_2$, 5 mM glucose and 10 mM HEPES, pH 7.4. The K^+ stimulation solution contained 30 mM KCl, 125 mM NaCl, 1.2 mM $MgCl_2$, 2 mM $CaCl_2$, 5 mM glucose, and 10 mM HEPES, pH 7.4. 10× Locke's buffer included 1540 mM NaCl, 56 mM KCl, 36 mM $NaHCO_3$, 56 mM glucose, 50 mM HEPES, 1% (v/v) penicillin, pH 7.4. This stock solution was diluted 10 times with distilled water (1× Locke's buffer) the day before the experiment.

2. Apparatus and Instruments.

2.1 Electrochemical measurements.

Before single cell amperometry (SCA) or intracellular vesicle impact electrochemical cytometry (IVIEC), the medium was removed and the cells were rinsed three times with the isotonic saline solution. The cells were kept at 37 °C in isotonic solution during the whole experimental process. Electrochemical recordings from single chromaffin cells were performed on an inverted microscope (IX71, Olympus), in a Faraday cage. The working potential was +700 mV versus an Ag/AgCl reference electrode (Scanbur, Sweden) under the control of an Axopatch 200B potentiostat (Molecular Devices, Sunnyvale, CA). The output was filtered at 2.1 kHz and digitized at 5 kHz (Axoscope 10.4 software, Axon Instruments Inc., Sunnyvale, CA, USA). All the experiments were observed under an inverted microscope (IX81, Olympus) with 10x and 40x objectives. For SCA, a disk carbon fiber microelectrode was moved slowly by a Patch-Clamp Micromanipulator (PCS-5000, Burleigh Instruments, Inc., USA) to place it on the membrane of a chromaffin cell without causing any damage to the surface. Ten seconds after the start of recording, 30 mM K^+ stimulating solution in a glass micropipette was injected into the surrounding of the chromaffin cells with a single 30-s injection pulse. For IVIEC, a nano-tip conical carbon fiber microelectrode was first placed on the top of a chromaffin cell. The tip was slowly pressed through the membrane of a chromaffin cell while the current was recorded. There is no need of stimulation in IVIEC. The data sampling rate was 10 kHz.

2.2 Data Acquisition and Analysis.

The amperometric traces were processed using an Igor Pro 6.22 routine originating from David Sulzer's group. The threshold for peak detection was three times the standard deviation of the noise. The traces were carefully inspected after peak detection and false positives were manually rejected. The number of molecules released by single cells was pooled, and the median of the data was calculated for each experimental condition. These

parameters (Figure S4), the rise time (t_{rise}), defined as the time separating 25% of the maximum from 75% of the maximum on the ascending part of the spike; the half peak width ($t_{1/2}$), defined as the width of the exocytotic at half of its magnitude; the fall time (t_{fall}), defined as the time separating 75% of the maximum from 25% of the maximum on the descending part of the spike. To compare between different conditions, the mean of medians of molecules number calculated was used. The responding cells were also calculated from each experiment. Statistical analysis was performed using one-way ANOVA with appropriate post-hoc testing with the Holm-Sidak method;[1] ***, $p < 0.001$; **, $p < 0.01$; *, $p < 0.05$.

3. Isolation of Adrenal Chromaffin Cells. Bovine adrenal glands were obtained from a local slaughterhouse, and the chromaffin cells were isolated as previously described.[2] Briefly, the vein was perfused with Locke's buffer to clear away blood cells. The medulla was isolated after collagenase (0.2%, Roche, Sweden) treatment, and cells were isolated using a series of homogenization and centrifugation steps. For single cell experiments, ~700 000 cells were seeded on collagen (IV) coated plastic dishes (D=60 mm, Corning Biocoat, VWR, Sweden) and maintained in a humidified incubator at 37 °C, 5% CO_2 for a maximum of 3 days prior to experiments. For different anions treatment, the cells were incubated with 100 μM X^- (e.g., Cl^-, Br^-, NO_3^-, ClO_4^-, and SCN^-) in media for 3 h before experiments. These values were chosen as typical serum levels of NO_3^- in the western world range from 10-140 μM, and the serum half-life is 5-8 h.[3] The serum Br^- levels range from 40-70 μM.[4] For SCN^-, nonsmokers' serum levels are typically 10-70 μM, compared to a typical range of 80-120 μM among smokers, it has a reported "kideney threshold" at a serum level of 200-300 μM, and the serum half-life is 3-6 days.[5,3c] Moreover, the effect of Zn^{2+} (kosmotropic cation) on the exocytosis and vesicular content has been investigated with these parameters (100 μM and 3.5 h Zn^{2+})[6], and exocytosis is regulated with Zn^{2+} (0.1, 1, 10, and 100 μM Zn^{2+})[7].

The ionic composition of the cell medium included 1.05 mM $CaCl_2$, 5.2E^{-6} mM $CuSO_4$, 1.24 mM $Fe(NO_3)_3$, 0.0015 mM $FeSO_4$, 0.30 mM $MgCl_2$, 0.407 mM $MgSO_4$, 4.16 mM KCl, 14.29 mM $NaHCO_3$, 120.6 mM NaCl, 0.50 mM Na_2HPO_4, 0.45 NaH_2PO_4, 0.0015 mM $ZnSO_4$.

4. Fabrication of Disk Carbon Fiber Microelectrodes. The fabrication of disk microelectrodes was previously described.[8] Briefly, a 5 μm diameter carbon fiber was aspirated into a glass capillary (Sutter Instrument Co., Novato, CA). A micropipette puller (model PE-21, Narishige, Inc., Japan) was used to pull the glass capillary into two separate electrodes and epoxy (Epoxy Technology, Billerica, MA, U.S.A.) was used to seal the electrodes. The glued electrodes were then cured at 100°C overnight and subsequently beveled at 45° angle (EG-400, Narishige Inc., London, UK). Before the experiment, each electrode was tested with cyclic voltammetry (-0.2 to 0.8 V vs Ag/AgCl,

100 mV/s) in a solution of 100 μM dopamine in PBS (pH 7.4). Only electrodes showing good reaction kinetics and stable steady-state currents were used for experiments.

5. Fabrication of Nano-Tip Conical Carbon Fiber Microelectrodes. The fabrication of nano-tip conical carbon fiber microelectrode was previously described.[9] Briefly, a 5 μm carbon fiber was aspirated into a borosilicate glass capillary (1.2 mm o.d., 0.69 mm i.d., Sutter Instrument Co., Novato, CA). The glass capillary was subsequently pulled into two separate electrodes with a commercial micropipette puller (model PE-21, Narishige, Inc., Japan). The fiber extending from the glass was cut to 100-150 μm with a scalpel under a microscope. To flame etch the carbon fiber, the electrodes were held on the edge of the blue part of a butane flame (Multiflame AB, Hässleholm, Sweden) for less than 2 s. As the end of the tip became red, the electrode was rotated in order to ensure even etching. Fibers with a needle-sharp tip about 50-100 nm tip diameter and about 30-100 μm shaft length were sealed with epoxy (Epoxy Technology, Billerica, MA). Each electrode was then tested by cyclic voltammetry (-0.2 to 0.8 V vs Ag/AgCl, 100 mV/s) in a solution of 100 μM dopamine in PBS (pH 7.4). Only electrodes showing good reaction kinetics and stable steady-state currents were used for experiments.

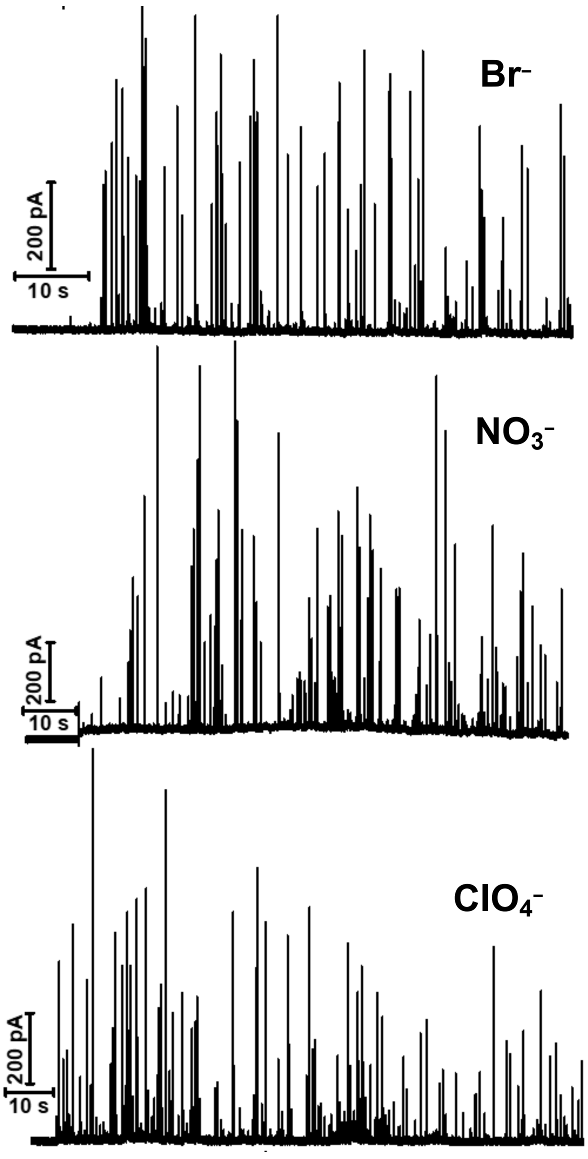

Figure S1. IVIEC amperometric traces obtained from chromaffin cells after exposure to 100 μM KX (X^-: Br^-, NO_3^-, ClO_4^-) for 3 h.

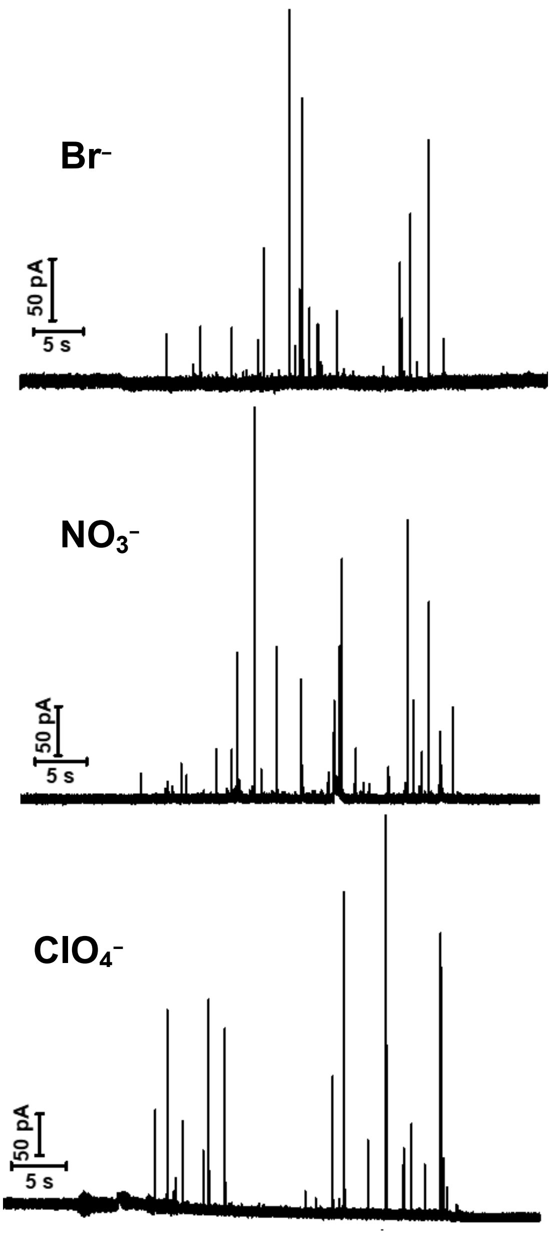

Figure S2. SCA amperometric traces obtained from chromaffin cells stimulated by 30 mM KCl solution after exposure to 100 μM KX (X^-: Br^-, NO_3^-, ClO_4^-) for 3 h.

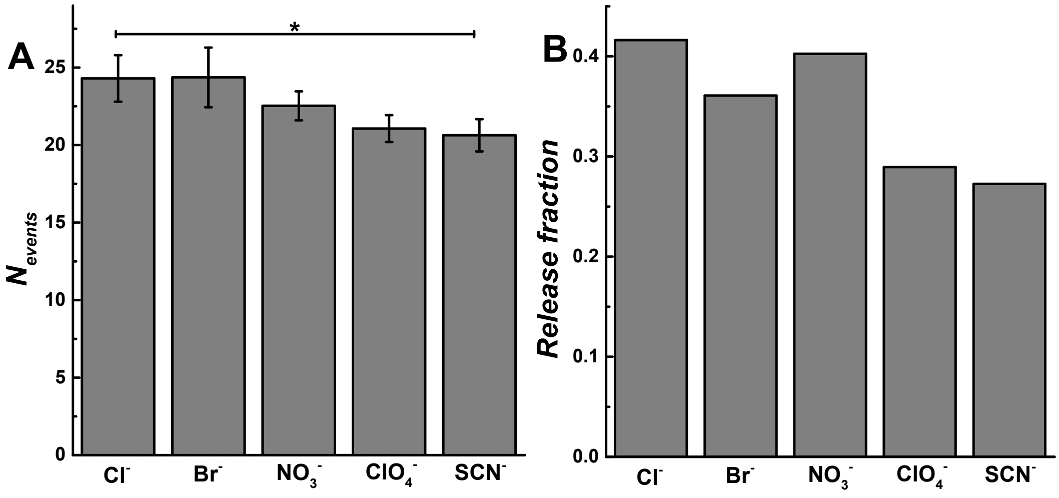

Figure S3. Comparisons of (A) number of exocytotic events, and (B) release fraction obtained from chromaffin cells which were stimulated by 30 mM KCl solution after exposure to 100 μM KX (X^-: Cl^-, Br^-, NO_3^-, ClO_4^-, SCN^-) for 3 h.

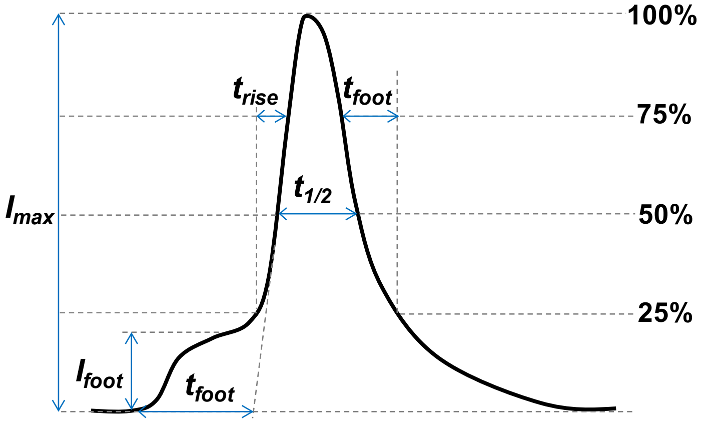

Figure S4. Schematic of the different parameters used for the peak analysis for exocytosis. I_{max}=peak current, t_{rise}=rise time, $t_{1/2}$=half peak width, t_{fall}=fall time, I_{foot}=foot current, t_{foot}=foot duration.

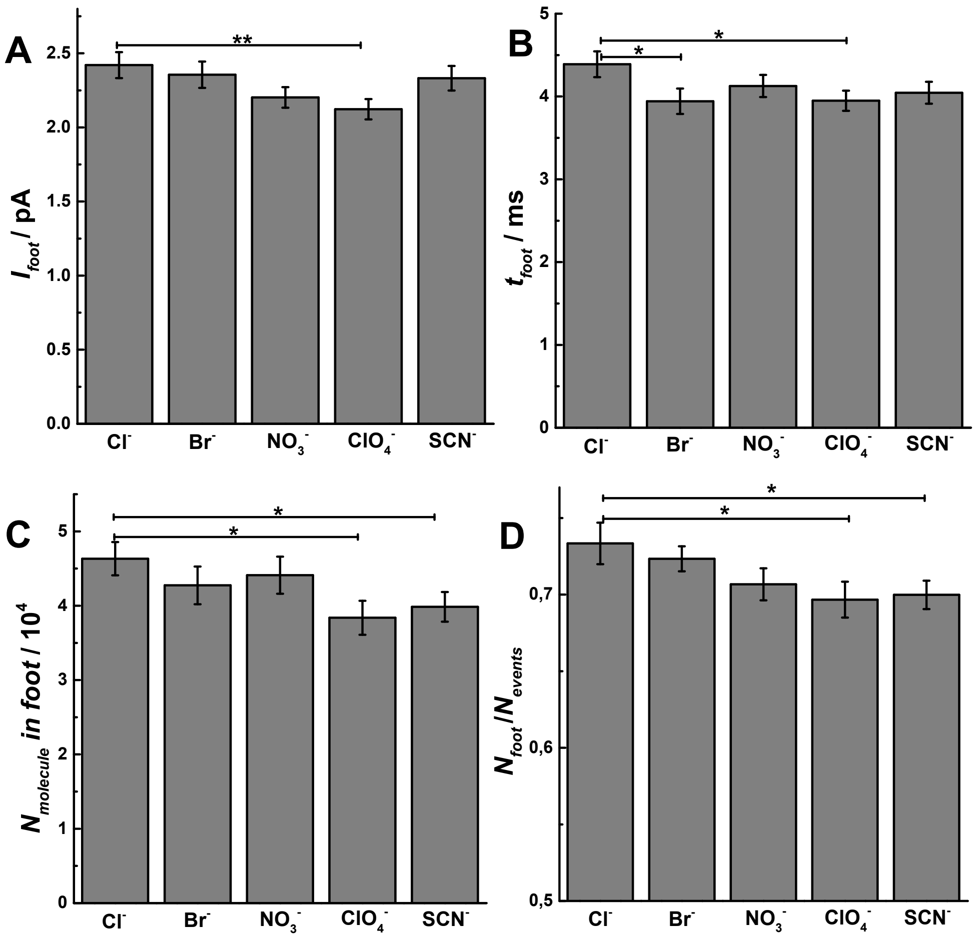

Figure S5. Foot parameters obtained from SCA obtained from chromaffin cells which were stimulated by 30 mM KCl solution after exposure to 100 μM KX (X^-: Cl^-, Br^-, NO_3^-, ClO_4^-, SCN^-) for 3 h.

Table S1. P values to compare $N_{molecules}$ obtained from IVIEC. N=12, $p < 0.05$, *; $p < 0.01$, **; $p < 0.001$, ***.

$N_{molecules}$	Cl^-	Br^-	NO_3^-	ClO_4^-	SCN^-
Cl^-	×	0.776<br>ns	0.281<br>ns	0.00106<br>**	0.000024<br>***
Br^-	0.776<br>ns	×	0.580<br>ns	0.0152<br>*	0.00219<br>**
NO_3^-	0.281<br>ns	0.580<br>ns	×	0.0182<br>*	0.00119<br>**
ClO_4^-	0.00106<br>**	0.0152<br>*	0.0182<br>*	×	0.501<br>ns
SCN^-	0.000024<br>***	0.00219<br>**	0.00119<br>**	0.501<br>ns	×

Table S2. P values to compare the number of event (N_{events}) obtained from SCA. N=30, $p < 0.05$, *.

N_{events}	Cl^-	Br^-	NO_3^-	ClO_4^-	SCN^-
Cl^-	×	0.978<br>ns	0.321<br>ns	0.0667<br>ns	0.0488<br>*
Br^-	0.978<br>ns	×	0.396<br>ns	0.124<br>ns	0.0935<br>ns
NO_3^-	0.321<br>ns	0.396<br>ns	×	0.256<br>ns	0.180<br>ns
ClO_4^-	0.0667<br>ns	0.124<br>ns	0.256<br>ns	×	0.750<br>ns
SCN^-	0.0488<br>*	0.0935<br>ns	0.180<br>ns	0.750<br>ns	×

Table S3. P values to compare number of molecules ($N_{molecules}$) obtained from SCA. N=30, $p < 0.05$, *; $p < 0.01$, **; $p < 0.001$, ***.

$N_{molecules}$	Cl^-	Br^-	NO_3^-	ClO_4^-	SCN^-
Cl^-	×	0.0351<br>*	0.0212<br>*	0.597<br>ns	0.728<br>ns
Br^-	0.0351<br>*	×	0.000033<br>***	0.0133<br>*	0.0342<br>*
NO_3^-	0.0212<br>*	0.000033<br>***	×	0.100<br>ns	0.114<br>ns
ClO_4^-	0.597<br>ns	0.0133<br>*	0.100<br>ns	×	0.906<br>ns
SCN^-	0.728<br>ns	0.0342<br>*	0.114<br>ns	0.906<br>ns	×

Table S4. P values to compare the peak parameter I_{max} obtained from SCA. N=30, $p < 0.05$, *; $p < 0.01$, **.

I_{max}	Cl^-	Br^-	NO_3^-	ClO_4^-	SCN^-
Cl^-	×	0.813<br>ns	0.0241<br>*	0.00188<br>**	0.00312<br>**
Br^-	0.813<br>ns	×	0.0244<br>*	0.00270<br>**	0.00329<br>**
NO_3^-	0.0241<br>*	0.0244<br>*	×	0.474<br>ns	0.237<br>ns
ClO_4^-	0.00188<br>**	0.00270<br>**	0.474<br>ns	×	0.506<br>ns
SCN^-	0.00312<br>**	0.00329<br>**	0.237<br>ns	0.506<br>ns	×

Table S5. P values to compare the peak parameter $t_{1/2}$ obtained from SCA. N=30, $p < 0.05$, *; $p < 0.01$, **; $p < 0.001$, ***.

$t_{1/2}$	Cl^-	Br^-	NO_3^-	ClO_4^-	SCN^-
Cl^-	×	0.758<br>ns	0.0358<br>*	0.0125<br>*	0.000101<br>***
Br^-	0.758<br>ns	×	0.0456<br>*	0.0144<br>*	0.000079<br>***
NO_3^-	0.0358<br>*	0.0456<br>*	×	0.614<br>ns	0.0121<br>*
ClO_4^-	0.0125<br>*	0.0144<br>*	0.614<br>ns	×	0.0344<br>*
SCN^-	0.000101<br>***	0.000079<br>***	0.0121<br>*	0.0344<br>*	×

Table S6. P values to compare the peak parameter t_{rise} obtained from SCA. N=30, $p < 0.05$, *.

t_{rise}	Cl^-	Br^-	NO_3^-	ClO_4^-	SCN^-
Cl^-	×	0.186<br>ns	0.137<br>ns	0.174<br>ns	0.0412<br>*
Br^-	0.186<br>ns	×	0.992<br>ns	0.861<br>ns	0.629<br>ns
NO_3^-	0.137<br>ns	0.992<br>ns	×	0.831<br>ns	0.586<br>ns
ClO_4^-	0.174<br>ns	0.861<br>ns	0.831<br>ns	×	0.423<br>ns
SCN^-	0.0412<br>*	0.629<br>ns	0.586<br>ns	0.423<br>ns	×

Table S7. P values to compare the peak parameter t_{fall} obtained from SCA. N=30, $p < 0.05$, *; $p < 0.01$, **.

t_{fall}	Cl^-	Br^-	NO_3^-	ClO_4^-	SCN^-
Cl^-	×	0.298<br>ns	0.00531<br>**	0.0293<br>*	0.0338<br>*
Br^-	0.298<br>ns	×	0.0397<br>*	0.168<br>ns	0.198<br>ns
NO_3^-	0.00531<br>**	0.0397<br>*	×	0.574<br>ns	0.464<br>ns
ClO_4^-	0.0293<br>*	0.168<br>ns	0.574<br>ns	×	0.889<br>ns
SCN^-	0.0338<br>*	0.198<br>ns	0.464<br>ns	0.889<br>ns	×

Table S8. P values to compare the peak parameter I_{foot} obtained from SCA. N=30, $p < 0.05$, *; $p < 0.01$, **.

I_{foot}	Cl^-	Br^-	NO_3^-	ClO_4^-	SCN^-
Cl^-	×	0.607<br>ns	0.0561<br>ns	0.00967<br>**	0.468<br>ns
Br^-	0.607<br>ns	×	0.179<br>ns	0.0424<br>*	0.847<br>ns
NO_3^-	0.0561<br>ns	0.179<br>ns	×	0.418<br>ns	0.235<br>ns
ClO_4^-	0.00967<br>**	0.0424<br>*	0.418<br>ns	×	0.0562<br>ns
SCN^-	0.468<br>ns	0.847<br>ns	0.235<br>ns	0.0562<br>ns	×

Table S9. P values to compare the peak parameter t_{foot} obtained from SCA. N=30, $p < 0.05$, *.

t_{foot}	Cl^-	Br^-	NO_3^-	ClO_4^-	SCN^-
Cl^-	×	0.0451<br>*	0.205<br>ns	0.0293<br>*	0.0978<br>ns
Br^-	0.0451<br>*	×	0.369<br>ns	0.973<br>ns	0.613<br>ns
NO_3^-	0.205<br>ns	0.369<br>ns	×	0.329<br>ns	0.668<br>ns
ClO_4^-	0.0293<br>*	0.973<br>ns	0.329<br>ns	×	0.593<br>ns
SCN^-	0.0978<br>ns	0.613<br>ns	0.668<br>ns	0.593<br>ns	×

Table S10 P values to compare $N_{molecules}$ in foot obtained from SCA. N=30, $p < 0.05$, *.

$N_{molecules}$ in foot	Cl^-	Br^-	NO_3^-	ClO_4^-	SCN^-
Cl^-	×	0.293<br>ns	0.512<br>ns	0.0158<br>*	0.0348<br>*
Br^-	0.293<br>ns	×	0.700<br>ns	0.207<br>ns	0.374<br>ns
NO_3^-	0.512<br>ns	0.700<br>ns	×	0.0952<br>ns	0.187<br>ns
ClO_4^-	0.0158<br>*	0.207<br>ns	0.0952<br>ns	×	0.630<br>ns
SCN^-	0.0348<br>*	0.374<br>ns	0.187<br>ns	0.630<br>ns	×

Table S11. P values to compare the percent of feet observed in all events (N_{foot}/N_{events}) obtained from SCA. N=30, $p < 0.05$, *.

N_{foot}/N_{events}	Cl^-	Br^-	NO_3^-	ClO_4^-	SCN^-
Cl^-	×	0.530<br>ns	0.124<br>ns	0.0451<br>*	0.0457<br>*
Br^-	0.530<br>ns	×	0.212<br>ns	0.0663<br>ns	0.0614<br>ns
NO_3^-	0.124<br>ns	0.212<br>ns	×	0.527<br>ns	0.626<br>ns
ClO_4^-	0.0451<br>*	0.0663<br>ns	0.527<br>ns	×	0.835<br>ns
SCN^-	0.0457<br>*	0.0614<br>ns	0.626<br>ns	0.835<br>ns	×

Table S12. P values to compare the peak parameter I_{max} obtained from IVIEC. N=12, $p < 0.05$, *.

I_{max}	Cl^-	Br^-	NO_3^-	ClO_4^-	SCN^-
Cl^-	×	0.158<br>ns	0.983<br>ns	0.0867<br>ns	0.322<br>ns
Br^-	0.158<br>ns	×	0.237<br>ns	0.941<br>ns	0.592<br>ns
NO_3^-	0.983<br>ns	0.237<br>ns	×	0.171<br>ns	0.422<br>ns
ClO_4^-	0.0867<br>ns	0.941<br>ns	0.171<br>ns	×	0.468<br>ns
SCN^-	0.322<br>ns	0.592<br>ns	0.422<br>ns	0.468<br>ns	×

Table S13. P values to compare the peak parameter $t_{1/2}$ obtained from IVIEC. N=12, $p < 0.05$, *; $p < 0.01$, **; $p < 0.001$, ***.

$t_{1/2}$	Cl^-	Br^-	NO_3^-	ClO_4^-	SCN^-
Cl^-	×	0.892<br>ns	0.443<br>ns	0.0326<br>*	0.000563<br>***
Br^-	0.892<br>ns	×	0.473<br>ns	0.0882<br>ns	0.00656<br>**
NO_3^-	0.443<br>ns	0.473<br>ns	×	0.213<br>ns	0.00904<br>**
ClO_4^-	0.0326<br>*	0.0882<br>ns	0.213<br>ns	×	0.0819<br>ns
SCN^-	0.000563<br>***	0.00656<br>**	0.00904<br>**	0.0819<br>ns	×

Table S14. P values to compare the peak parameter t_{rise} obtained from IVIEC. N=12, $p < 0.05$, *; $p < 0.01$, **; $p < 0.001$, ***.

t_{rise}	Cl^-	Br^-	NO_3^-	ClO_4^-	SCN^-
Cl^-	×	0.886<br>ns	0.732<br>ns	0.107<br>ns	0.000009<br>***
Br^-	0.886<br>ns	×	0.893<br>ns	0.348<br>ns	0.00143<br>**
NO_3^-	0.732<br>ns	0.893<br>ns	×	0.342<br>ns	0.000343<br>***
ClO_4^-	0.107<br>ns	0.348<br>ns	0.342<br>ns	×	0.000070<br>***
SCN^-	0.000009<br>***	0.00143<br>**	0.000343<br>***	0.000070<br>***	×

Table S15. P values to compare the peak parameter t_{fall} obtained from IVIEC. N=12, $p < 0.05$, *; $p < 0.01$, **; $p < 0.001$, ***.

t_{fall}	Cl^-	Br^-	NO_3^-	ClO_4^-	SCN^-
Cl^-	×	0.949<br>ns	0.999<br>ns	0.0457<br>*	0.000295<br>***
Br^-	0.949<br>ns	×	0.954<br>ns	0.0755<br>ns	0.00186<br>**
NO_3^-	0.999<br>ns	0.954<br>ns	×	0.0793<br>ns	0.00178<br>**
ClO_4^-	0.0457<br>*	0.0755<br>ns	0.0793<br>ns	×	0.130<br>ns
SCN^-	0.000295<br>***	0.00186<br>**	0.00178<br>**	0.130<br>ns	×

References

(1) a) Dinno, A. *Stata J.* **2015**, *15*, 292−300; b) Nguyen, T. T.; Huynh, N. N-C.; Seubbuk, S.; Nilmoje, T.; Wannasuntronwong, A.; Surarit, R. *Odontology* **2019**, *107*, 133−141; c) Aggarwal, V.; Singla, M.; Sharma, R.; Miglani, S.; Bhasin, S. S. *J. Conserv. Dent.* **2016**, *19*, 419−423.

(2) O'Connor, D. T.; Mahata, S. K.; Mahata, M.; Jiang, Q.; Hook, V. Y.; Taupenot, L. *Nat. Protoc.* **2007**, *2,* 1248−1253.

(3) a) Kassim, S. K.; El Touny, M.; El Guinaidy, M.; El Moghni, M. A.; El Mohsen, A. A. *Clin. Biochem.* **2002**, *35*, 641−646；b) Watanabe, T.; Akishita, M.; Toba, K.; Kozaki, K.; Eto, M.; Sugi moto, N.; Kiuchi, T.; Hashimoto, M.; Shirakawa, W.; Ouchi, Y. *Clin. Chim. Acta.* **2000**, *301*, 169−179; c) Tonacchera, M.; Pinchera, A.; Dimida, A.; Ferrarini, E.; Agretti, P.; Vitti, P.; Santini, F.; Crump, K.; Gibbs, J. *Thyroid* **2004**, *14*, 1012−1019.

(4) Cuenca, R. E.; Pories, W. J.; Bray, J. *Biol. Trace Elem. Res.* **1988**, *16*, 151−154.

(5) a) Foss, O. P.; Lund-larsen, P. G. *Scand. J. Clin. Lab. Invest.* **1986**, *46*, 245−251; b) Robertson, A. S.; Burge, P. S.; Cockrill, B. L. *Br J. Ind. Med.* **1987**, *44*, 351−354; c) Chen, Y.; Pederson, L. L. Lefcoe, N. M. *Arch. Environ. Health* **1990**, *45*, 163−167; d) Schulz, V.; Bonn, R.; Kindler, J. *Klin. Wochenschr.* **1979**, *57*, 243−247.

(6) Ren, L.; Pour, M. D.; Majdi, S.; Li, X.; Malmberg, P.; Ewing, A. G. A*ngew. Chem. Int. Ed.* **2017**, *56*, 4970−4975.

(7) Ren, L.; Pour, M. D.; Malmberg, P.; Ewing, A. G. *Chem. Eur. J.* **2019**, *25*, 5406−5411

(8) Adams, K. L.; Engelbrektsson, J.; Voinova, M.; Zhang, B.; Eves, D. J.; Karlsson, R.; Heien, M. L.; Cans, A. S.; Ewing, A. G. *Anal. Chem.* **2010**, *82*, 1020−1026.

(9) a) Strein, T. G.; Ewing, A. G. *Anal. Chem.* **1992**, *64*, 1368−1373; b) Kawagoe, K. T.; Jankowski, J. A.; Wightman, R. M. *Anal. Chem.* **1991**, *63*, 1589−1594; c) Strand, A. M.; Venton, B. J. *Anal. Chem.* **2008**, *80*, 3708−3715.
